# Tick-Tattoo: DNA Vaccination Against *B. burgdorferi* or *Ixodes scapularis* Tick Proteins

**DOI:** 10.3389/fimmu.2021.615011

**Published:** 2021-02-25

**Authors:** Michelle J. Klouwens, Jos J. A. Trentelman, Alex Wagemakers, Jasmin I. Ersoz, Adriaan D. Bins, Joppe W. Hovius

**Affiliations:** ^1^ Department of Internal Medicine, Center for Experimental and Molecular Medicine, Academic Medical Center, University of Amsterdam, Amsterdam, Netherlands; ^2^ Division of Infectious Diseases, Department of Internal Medicine, Academic Medical Center, Amsterdam, Netherlands; ^3^ Amsterdam Multidisciplinary Lyme Borreliosis Center, Academic Medical Center, Amsterdam, Netherlands

**Keywords:** lyme disease, *borrelia*, DNA tattoo, DNA vaccination, OspC, tick proteins

## Abstract

**Introduction:**

*Borrelia burgdorferi* sensu lato (sl) is the causative agent of Lyme borreliosis. Currently there is no human vaccine against Lyme borreliosis, and most research focuses on recombinant protein vaccines. DNA tattoo vaccination with *B. afzelii* strain PKo OspC in mice has proven to be fully protective against *B. afzelii* syringe challenge and induces a favorable humoral immunity compared to recombinant protein vaccination. Alternatively, several recombinant protein vaccines based on tick proteins have shown promising effect in tick-bite infection models. In this study, we evaluated the efficacy of DNA vaccines against *Borrelia* OspC or tick antigens in a tick-bite infection model.

**Method:**

We vaccinated C3H/HeN mice with OspC using a codon-optimized DNA vaccine or with recombinant protein. We challenged these mice with *B. burgdorferi* sensu stricto (ss)-infected *Ixodes scapularis* nymphs. Subsequently, we vaccinated C3H/HeN mice with DNA vaccines coding for tick proteins for which recombinant protein vaccines have previously resulted in interference with tick feeding and/or *Borrelia* transmission: Salp15, tHRF, TSLPI, and Tix-5. These mice were also challenged with *B. burgdorferi* ss infected *Ixodes scapularis* nymphs.

**Results:**

DNA tattoo and recombinant OspC vaccination both induced total IgG responses. *Borrelia* cultures and DNA loads of skin and bladder remained negative in the mice vaccinated with OspC DNA vaccination, except for one culture. DNA vaccines against tick antigens Salp15 and Tix-5 induced IgG responses, while those against tHRF and TSLPI barely induced any IgG response. In addition, *Borrelia* cultures, and DNA loads from mice tattooed with DNA vaccines against tick proteins TSLPI, Salp15, tHRF, and Tix-5 were all positive.

**Conclusion:**

A DNA tattoo vaccine against OspC induced high specific IgG titers and provided near total protection against *B. burgdorferi* ss infection by tick challenge. In contrast, DNA tattoo vaccines against tick proteins TSLPI, Salp15, tHRF, and Tix-5 induced low to moderate IgG titers and did not provide protection. Therefore, DNA tattoo vaccination does not seem a suitable vaccine strategy to identify, or screen for, tick antigens for anti-tick vaccines. However, DNA tattoo vaccination is a straightforward and effective vaccination platform to assess novel *B. burgdorferi* sl antigen candidates in a relevant tick challenge model.

## Introduction

Lyme borreliosis is the most common vector-borne disease in the Northern hemisphere and is caused by the spirochete *Borrelia burgdorferi* sl, which is transmitted by *Ixodes* ticks. Vaccination would be an effective way to prevent Lyme disease. Currently there is no human vaccine available. Vaccines to prevent *Borrelia burgdorferi* sl infection could work in two ways: killing the pathogen to stop infection or targeting the vector to prevent successful transmission. Research therefore focuses on either protective antigens derived from the pathogen, *B. burgdorferi* sl, or from the vector, *Ixodes* ticks (1). When focusing on possible protective antigens from *Borrelia*, the most promising candidates in human vaccine studies are the outer surface proteins. Especially OspA, which is primarily expressed by *Borrelia* in unfed ticks, has been widely studied and was the primary component of the withdrawn human LYMErix™ vaccine (2–6). During transmission from tick to host, the *Borrelia* spirochete downregulates OspA and upregulates outer surface protein C, which is necessary to facilitate migration to the tick salivary glands and also plays a role in spirochete infection of the mammalian host. OspC was also shown to be an effective vaccine target, but has a high heterogenicity among different *B. burgdorferi* sl species and strains (7, 8).

In an alternative approach where the tick vector is targeted, tick saliva could play a pivotal role. Tick saliva contains several proteins that facilitate transmission and survival of tick-borne pathogens by using anti-inflammatory, anti-coagulant and immunosuppressive abilities (9, 10). *Borrelia burgdorferi* sl exploits tick salivary gland proteins to facilitate their transmission from tick to host, and vice versa to increase their chances of survival within the tick (11, 12). For example, OspC binds to *Ixodes scapularis* salivary protein Salp15 which protects the spirochete from antibody-mediated killing (12–14). In addition, Salp15 also has immunosuppressive properties in inhibiting CD4^+^ T cell and dendritic cell activation (15, 16). Interestingly, a vaccine directed against Salp15 has been shown to partially block *B. burgdorferi* ss infection (14, 17). Dai et al. also characterized tick histamine release factor, present in tick saliva and important to tick feeding (18). They showed significantly impaired tick feeding on mice when tHRF was silenced by RNA interference. Tick feeding and transmission of *B. burgdorferi* ss was also significantly diminished in tHRF immunized mice (18). Schuijt et al. identified Tick Salivary Lectin Pathway Inhibitor (TSLPI), an *I. scapularis* salivary protein, which was shown to impair complement-mediated killing of *B. burgdorferi. B. burgdorferi* transmission was impaired in mice that were injected with TSLPI rabbit antiserum (19). TIX-5 (tick inhibitor of factor Xa toward factor V) is another tick protein, with anticoagulant activity, that has been investigated in vaccination studies (20, 21). Adult *I. scapularis* tick engorgement weights from rTIX-5–immune rabbits were dramatically reduced compared to control rabbits. The effect on *B. burgdorferi* ss transmission has not been assessed. Thus, multiple promising tick salivary gland proteins have been identified and investigated as vaccine candidates to prevent tick feeding and/or transmission of *B. burgdorferi* sl from tick to host. It has been described in literature that these salivary gland antigens are expressed in infected ticks; Salp15 and TSLPI are even upregulated in infected ticks (13, 19). In addition, transcriptomic data from *B. afzelii* infected *I. ricinus* nymphs show that all these antigens are also expressed in infected *I. ricinus* salivary glands, indicating that the expression of these specific antigens appears particularly conserved even cross-species (22).

In addition to *B. burgdorferi* sl, *Ixodes* ticks also transmit other tick-borne diseases that can cause human infection such as Babesiosis and Anaplasmosis and several Flaviviruses. An anti-tick vaccine that would prevent the tick from feeding on a host could have the advantage of being able to provide protection against multiple tick-borne diseases (23).

Most research on new Lyme vaccines focuses on recombinant proteins, but DNA vaccination constitutes an alternative vaccination platform (24). DNA vaccines are easy to produce, highly stable and induce both humoral and cellular immune responses (25). While no human genomic vaccines targeting infectious diseases are currently on the market, the COVID pandemic might establish its mainstream acceptance in infectious diseases, as several genomic vaccines are currently being developed (26–28). A previous study by Wagemakers et al. has shown that DNA vaccination by tattoo with OspC from *B. afzelii* strain PKo as a model antigen was fully protective against *B. afzelii* syringe challenge in mice and induced favorable humoral immune responses compared to recombinant protein vaccination (29). In the current study, we have used OspC from *B. burgdorferi* ss strain N40, both as recombinant as well as DNA vaccine to evaluate whether DNA vaccination can also protect against *Borrelia* infection through tick challenge, more closely resembling the natural situation. The other goal of this study was to assess DNA vaccination as a modality to induce protective immune responses against tick antigens. We assessed tick salivary gland proteins TSLPI, Salp15, tHRF, and TIX-5 to test DNA vaccination as an easy screening vaccination platform for novel future candidates as an anti-tick vaccine. These described tick antigens were selected since they are known to be able to interfere with tick feeding and/or *B. burgdorferi* transmission when investigated in conventional vaccination approaches.

## Materials and Methods

### Ethics Statement

All experiments were reviewed and approved by the Animal Research Ethics Committee of the Academic Medical Center, Amsterdam, The Netherlands (protocol 208AI). Experiments have been conducted according to European and national guidelines.

### Recombinant OspC Protein Generation

The OspC gene was amplified from genomic DNA from *Borrelia burgdorferi* ss strain N40 DNA and was cloned into pET-21b (Invitrogen), produced in *E. coli* and purified using Ni-NTA as detailed elsewhere (12).

Purity was checked using SDS-PAGE, and protein concentrations were measured using a Bradford assay.

### Generation of DNA Vaccines

The DNA vaccines were designed as described before in Wagemakers et al. (29). From *the B. burgdorferi* N40 OspC gene sequence (NCBI reference DQ437463.1 and the respective tick salivary gland genes Salp15 (NCBI reference AAK97817.1), tHRF (NCBI reference DQ066335), TSLPI (NCBI reference AEE89466.1), and TIX-5 (NCBI reference AEE89467**).** The signal peptide (predicted by SignalP 4.0 web-based software, CBS, Lyngby, Denmark) was replaced with the human tissue plasminogen activator (hTPA) signal sequence (genbank AAA61213.1) (30). The resulting sequence was codon-optimized to mouse tRNA usage with Java Codon Adaptation tool (Braunschweig, Germany) (31). At the 5′ end a BamH1 and a Kozak sequence were added, and at the 3′ end a sequence encoding a double stop codon and a Xho1 were added. The insert was synthesized (BaseClear, Leiden, The Netherlands) and ligated into a BamH1/Xho1 restricted empty pVAX vector (Invitrogen, Carlsbad, CA, USA). The plasmid was amplified using a Nucleobond Xtra EF kit (Macherey-Nagel, Düren, Germany) and resuspended in DNase free water.

### Generation of I. scapularis Nymphs with *B. burgdorferi* Strain N40

Low passage *B. burgdorferi* ss strain N40 spirochetes were cultured in MKP medium and counted by using a Petroff-Hausser counting chamber and dark-field microscopy. 1x10^6^ spirochetes in 200 µl was injected subcutaneously between the shoulders of four six-to-eight-weeks-old female C3H/HeN mice, purchased from Charles River. Mice were checked for *Borrelia* infection positivity by qPCR after 14 days. Once infection was confirmed, approximately 500 *I. scapularis* larvae (kindly provided by Center for Disease Control and Prevention, BEI Resources, NIAID, NIH: *Ixodes scapularis* (Live), NR-44116) were placed on each mouse. In the following 6 days, the fully fed larvae that had fallen off the mice were collected and were allowed to mold into the nymphal stage during the next 6–8 weeks. Ticks were housed in an incubator (Panasonic) at room temperature and at a constant relative humidity of 90%. Once molted, nymphal infection rates were assessed by qPCR. To establish tick infection rate, DNA was extracted from 10 ticks using the Qiagen Blood and Tissue kit (Qiagen, Venlo, The Netherlands). Quantitative (q)PCR was used to quantify *B. burgdorferi* ss DNA in mouse tissues and was performed according to previously described protocol (29) and also in the section below “Borrelia detection and quantification by culture and qPCR”. Infection rate was > 90%.

### Vaccination Experiments

Six-to-eight-weeks-old female C3H/HeN mice were purchased from Charles River. The vaccination experiment was carried out as previously described (29). Eight mice per experimental group were vaccinated at t=0, t=14, and t=28 days and sera were collected at each time point. For the recombinant OspC vaccine 10 μg protein was emulsified with complete Freund’s adjuvant at t=0 and 5 μg in incomplete Freund’s adjuvant at t=14 and t=28 days. All vaccinations were administered subcutaneously. For the DNA vaccines and the negative control, hair was removed from the mouse abdomen using hair removal cream. Using a Cheyenne Hawk tattoo machine carrying a Cheyenne 13-magnum tattoo needle (both MT.DERM, Berlin, Germany) 20 μg of the DNA vaccines was tattooed 0.5–1mm into the abdominal skin of the mice for 45 s at 100 Hz under isofluorane anesthesia. Two weeks after the third vaccination, at t=42, all mice were challenged with seven *Ixodes scapularis* nymphs, infected with *B. burgdorferi ss* strain N40. To determine tick attachment time and tick weights the *B. burgdorferi* ss strain N40-infected *I. scapularis* nymphs were placed in capsules on the vaccinated mice and allowed to feed to repletion. The nymphs were checked daily for attachment, collected and weighed when they had fallen off. Additional sera were collected at t=42 (pre-challenge) and at t=63 days mice were sacrificed and ear, skin, ankle, heart, bladder, and tissue was collected for analysis.

### ELISA

To measure antigen-specific IgG, ELISAs were performed, as described previously (29). High-binding 96-well ELISA plates (Greiner Bio-one, Kremsmünster, Austria) were coated overnight at 4°C with 1 μg/ml recombinant protein (produced as described elsewhere (12, 18, 19, 21)), washed with PBS–Tween (phosphate-buffered saline–0.05% Tween) and incubated with blocking buffer (1% BSA in PBS, pH 6.5) for 2 h at room temperature. Mouse sera (collected at day 42 before tick challenge) were diluted in blocking buffer, added to the wells and incubated for 1 h at room temperature. Plates were washed and incubated for 1 h with horseradish peroxidase (HRP)-linked anti-mouse IgG (Cell Signaling, Beverly, MA, USA) diluted 1:1,000 in blocking buffer. The plates were washed again and developed using TMB substrate [50 µl TMB chromogene in 5 ml TMB substrate buffer (8,2 gr NaAc and 21 gr citric acid monohydrate dissolved in 1 liter H_2_O + 10 µl 3% H_2_O_2_, pH 5)] and optical density was measured in a Biotek (Winooski, VT, USA) ELISA plate reader at 450–655 nm.

### Borrelia Detection and Quantification by Culture and qPCR

Cultures were carried out as described elsewhere (12). Murine bladder and skin samples were cultured in modified Kelly Pettenkofer (MKP) medium with rifampicin, 50 μg/ml and phosphomycin, 100 μg/ml) at 33°C. The cultures were checked weekly (for a total of 8 weeks) for the presence of motile spirochetes with dark field microscopy as described before (12). For all samples DNA was extracted using Qiagen Blood and Tissue kit (Qiagen, Venlo, The Netherlands). Quantitative (q)PCR was used to quantify *B. burgdorferi* ss DNA in mouse tissues and was performed according to previously described protocol (29). OspA primers were used for quantification; forward 5’-AAAAATATTTATTGGGAATAGGTCT-3’ and reverse 5’-CACCAGGCAAATCTACTGAA-3’, mouse Beta-actin forward 5’-AGCGGGAAATCGTGCGTG-3’ and reverse primer 5’-CAGGGTACATGGTGGTGCC-3’ were used for normalization. The qPCRs were performed on the LightCycler480 (Roche, Nutley, NJ, USA) using SYBR green dye (Roche) using the following PCR protocol: 95°C 6 min, and 60 cycles of 95°C 10 s, 60°C 20 s, and 72°C 20 s. Reactions were performed in triplicate. Results were analyzed using LinRegPCR software (Amsterdam, The Netherlands) (32). Negative and positive controls were included in each qPCR run.

### Statistical Methods

Differences between experimental groups between *B. burgdorferi* ss loads in qPCR were statistically tested by two-sided nonparametric tests (Mann-Whitney, GraphPad Prism software version 5.0, San Diego, CA, USA). Differences between experimental groups in tick weight were statistically tested by one-way ANOVA.

## Results

### Osp C Vaccination

**Figure 1:**
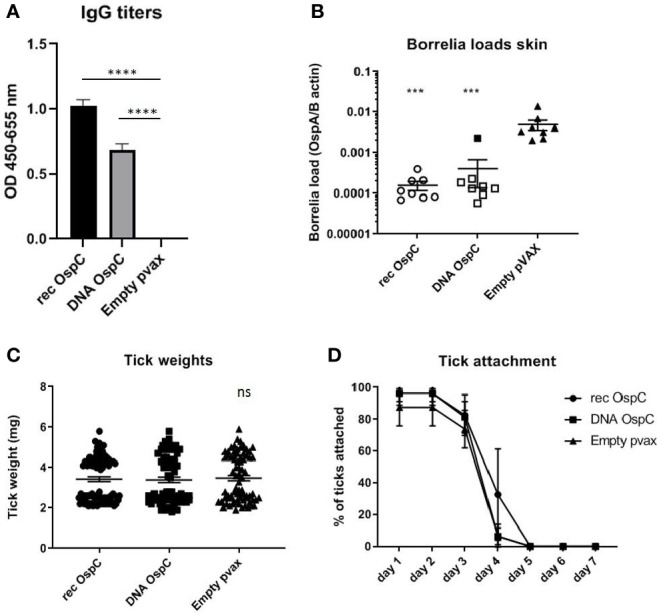
**(A–D)** rOspC vaccination versus vaccination with OspC DNA vaccine in a 0-14-28 day immunization protocol. **(A)** Specific total IgG titers were measured in an Enzyme-linked immunosorbent assay (ELISA). Plates were coated with recombinant protein and incubated with mouse sera collected at timepoint 42 days pre-challenge. Sera was diluted in steps of 3 starting with 100 times and ending with 218,700 times dilution Presented are the total IgG titers incubated with mouse sera 1:8,100 diluted for OspC. Statistical significance was calculated for each experimental group compared to the control, Empty pvax, using a one-way ANOVA statistical test (p< 0.05, **p<0.01, ***p<0.001, ****p<0.0001). **(B)** Borrelia loads in skin samples of the tick attachment site as determined by qPCR. Closed dots depict positive Borrelia loads, open dots depict PCR negative samples for which the OspA detection limit was divided by the sample’s mouse beta actin load. The Borrelia loads were compared to the negative control Empty pvax using a two-sided non parametric test (Mann-Whitney *P < 0.05, **P<0.01, ***P<0.001). **(C)** Tick weights in mg. Immunized mice were challenged with *B. burgdorferi* N40-infected I. scapularis nymphs. Nymphs were placed in a collection capsule on the back of each mice. The capsules were checked daily and the ticks were allowed to feed to repletion. Once they had fallen off their weight was measured. **(D)** Tick attachment is presented as percentage of ticks that are still attached per day. ns, not significant.

Vaccination with recombinant OspC as a positive control was compared to vaccination with OspC as a DNA vaccine to determine the efficacy of the DNA vaccine strategy (**Figure 1**). Both the rOspC and OspC DNA vaccine were able to induce robust IgG responses, although the titer of rOspC was significantly higher (**Figure 1A**). As expected from vaccination with a *Borrelia* protein, the OspC vaccinated groups did not show decreased tick weight or duration of attachment (**Figures 1C, D**).

We also assessed whether the conventional and DNA OspC vaccines were able to provide protection against infection with *B. burgdorferi ss* strain N40 transmitted by *I. scapularis* nymphs. For this purpose we performed qPCR and culture of several tissues we obtained by sacrificing the mice 21 days after the challenge. *B. burgdorferi* ss DNA loads in the skin challenge site were negative in all of the rOspC vaccinated mice and in seven out of eight of OspC DNA vaccinated mice (**Figure 1B**). *B. burgdorferi* ss culture data of the skin challenge site and bladder, corresponded with the qPCR data; seven out of eight mice were *Borrelia* negative (**Table 1**).

**Table 1:** Culture positivity 8 weeks after challenge for OspC vaccination.

	Skin	Bladder
Rec OspC	0/8	0/8
DNA OspC	1/8	1/8
Empty pvax	8/8	8/8

Cultures of skin and bladder were checked weekly for growth of Borrelia.

### Tick Salivary Gland DNA Vaccines

**Figure 2:**
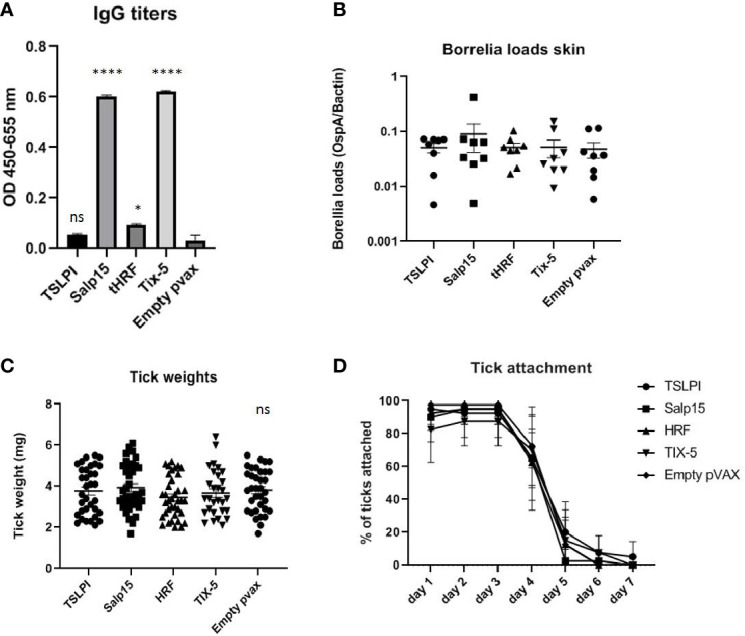
**(A–D)** DNA vaccination with tick salivary gland genes TSLPI, tHRF, Salp15, and Tix-5 in a 0-14-28 day immunization protocol. **(A)** Specific total IgG titers were measured in an Enzyme-linked immunosorbent assay (ELISA). Plates were coated with recombinant protein and incubated with mouse sera collected at timepoint 42 days pre-challenge. Sera was diluted in steps of 3 starting with 100 times and ending with 218,700 times dilution. Presented are the total IgG titers incubated with mouse sera 1:300 diluted for the tick salivary gland gene DNA vaccines. Statistical significance was calculated for each experimental group compared to the control, Empty pvax, using a one-way ANOVA statistical test (p< 0.05, **p<0.01, ***p<0.001, ****p<0.0001). **(B)** Borrelia loads in skin samples of the tick attachment site as determined by qPCR. Closed dots depict positive Borrelia loads, open dots depict PCR negative samples for which the OspA detection limit was divided by the sample’s mouse beta actin load. The Borrelia loads were compared to the negative control Empty pvax using a two-sided non parametric test (Mann-Whitney *P < 0.05, **P<0.01, ***P<0.001). **(C)** Tick weights in mg. Immunized mice were challenged with *B. burgdorferi* N40-infected I. scapularis nymphs. Nymphs were placed in a collection capsule on the back of each mice. The capsules were checked daily and the ticks were allowed to feed to repletion. Once they had fallen off their weight was measured. **(D)** Tick attachment is presented as percentage of ticks that are still attached per day. ns, not significant.

DNA vaccines against TSLPI, Salp15, tHRF, and Tix-5 did not induce robust IgG responses (**Figure 2A**). Although moderate antibody response against Salp 15 and Tix-5 compared to Empty pvax could be observed, TSLPI and tHRF did not elicit any noteworthy IgG response. When assessing tick attachment time and tick weight, the DNA vaccines against tick antigens also did not demonstrate a difference compared to the Empty pvax control (**Figures 2C, D**). As described above, OspC DNA vaccination protected all but one mouse against *Borrelia* infection. In stark contrast, *B. burgdorferi* ss DNA loads in TSLPI, Salp15, tHRF, and Tix-5 skin samples, were all positive (**Figure 2B**). Also, *B. burgdorferi* ss cultures from mice tattooed with DNA vaccines against tick proteins TSLPI, Salp15, tHRF, and TIX-5 were all positive and almost all within 2 weeks time (**Table 2**).

**Table 2:** Culture positivity 8 weeks after challenge for tick salivary gland DNA vaccination.

	Skin	Bladder
TSLPI	8/8	8/8
Salp15	8/8	8/8
tHRF	8/8	8/8
Tix-5	8/8	8/8
Empty pvax	8/8	8/8

Cultures of skin and bladder were checked weekly for growth of Borrelia.

## Discussion

In this study, we assessed the utility of DNA vaccination against Lyme borreliosis using a known *Borrelia* antigen - OspC - that has been shown to elicit protection when used as a recombinant protein vaccine. Both rOspC and OspC DNA vaccination resulted in robust antibody production in mice and subsequently protected against *B. burgdorferi* ss transmission by tick challenge. DNA vaccination therefore embodies a promising vaccination strategy, as it allows for rapid vaccination schedules, they are easy to produce, and besides humoral immunity, are also capable of inducing cellular immunity. Especially humoral immunity is described to be very important for clearance of *Borrelia* (33, 34). Interestingly, as the OspC DNA vaccine was able to induce moderate to high antibody titers and provided near total protection against *B. burgdorferi* ss infection by tick challenge. Therefore, immunization with plasmid DNA for this particular *Borrelia* antigen is, as described previously, an alternative platform for vaccination, as opposed to recombinant protein vaccination (35). It also shows that DNA vaccination against *B. burgdorferi* ss antigens in itself is effective in protecting against infection in a tick challenge model. Given the heterogeneity of OspC protein sequences among different *B. burgdorferi* sl species and strains, DNA vaccination could be an interesting platform, as multiple OspC sequence for a multivalent vaccine can be relatively easily combined (36). Future studies should focus on the effectiveness of a DNA vaccine targeting various OspC variants on protection against a range of *B. burgdorferi* sl isolates.

Secondly, we examined several anti-tick vaccine candidates as DNA vaccines. The ability of *Borrelia* spirochetes to establish an infection in mammals is partly dependent on tick (salivary gland) proteins, which makes these proteins interesting candidates for an anti-tick vaccine. Our goal was to determine the protective abilities of four tick salivary gland proteins used as DNA vaccines. In contrast to their recombinant protein vaccines described in literature, the tick salivary gland antigen DNA vaccines induced low (Salp15 and Tix-5) or hardly any (TSLPI and tHRF) IgG titers and did not provide protection against *B. burgdorferi* ss infection by tick challenge. It could therefore be speculated that the low or absent IgG titers are responsible for the fact that there was no protection observed and an adequate humoral immune response is essential to neutralize the function of these tick proteins and preventing transmission of *B. burgdorferi* ss. The low expression of these antigens by murine cells could be one reason for the low immunogenicity. Perhaps this could be circumvented by stronger adjuvants. Indeed, in addition to our strategy - i.e., addition of a hTPA signal and Kozak sequence and codon-optimization - adjuvant modifications can be made to DNA vaccines, such as genetic adjuvant strategies to improve the immune response induced by DNA vaccines (37). Regardless, both Salp15 and TIX5 were able to elicit a moderate IgG response, yet no protection against *B. burgdorferi* infection was observed. It should be mentioned that, although it has been established that an anti-tick vaccine based on TIX-5 impairs tick-feeding the effect of such a vaccine on *Borrelia* transmission has never been investigated (21). In contrast, for Salp15, for which an adjuvanted recombinant protein vaccine was able to interfere with *B. burgdorferi* transmission, the observed low IgG titers induced by our DNA vaccine are likely to cause for the lack of protection.

In this study, we have assessed DNA vaccination as a tool for two different vaccination approaches to protect against *B. burgdorferi* ss infection: targeting the spirochete with OspC or targeting the tick vector using Salp15, Tix-5, TSLPI, or tHRF. Although IgG levels are important for both approaches, the mechanism that leads to protection as a result of these antibodies differ greatly. Antibodies bound to OspC not only neutralize the ability of OspC to interact with Salp15 and shield against complement-mediated killing, they facilitate complement-mediated killing and phagocytosis of the spirochete. Antibodies against tick salivary gland proteins that are not directly bound to *Borrelia* can only neutralize the function of these proteins. As such high IgG levels against tick salivary gland proteins might possibly even be more important in protection compared to *Borrelia* antigens. Regardless, and as discussed above, it is clear that IgG levels induced by DNA vaccination against these tick proteins are insufficient (14).

In conclusion, we have shown that a successful vaccine against *Borrelia* is not restricted to conventional recombinant vaccination strategies and also works against tick-mediated transmission. This implies that DNA tattoo vaccination can be used to as a rapid and relatively easily screening tool to assess immunogenicity and efficacy of future novel *B. burgdorferi* sl vaccine candidates. In contrast, DNA vaccination appears not to be a suitable method to induce adequate immune responses against tick antigens and subsequent protection against *B. burgdorferi* ss; at least not for the selected tick salivary gland antigens Salp15, tHRF, TSLPI, and TIX-5.

## Data Availability Statement

The raw data supporting the conclusions of this article could be made available upon reasonable request.

## Ethics Statement

The animal study was reviewed and approved by Animal Research Ethics Committee of the Academic Medical Center, Amsterdam, The Netherlands.

## Author Contributions

MK, JT, and AW designed the study. JE, JT, and MK performed the study procedures. AB and AW provided information about the DNA vaccine design. JH supervised the study progress. All authors contributed to the article and approved the submitted version.

## Funding

This project has received funding from the European Union’s Seventh Programme for research, technological development, and demonstration under grant agreement No. 602272 of which JH was the scientific coordinator.

## Conflict of Interest

The authors declare that the research was conducted in the absence of any commercial or financial relationships that could be construed as a potential conflict of interest.

## Supplementary Material

Supplementary Figure 1 Specific total IgG titers were measured in an Enzyme-linked immunosorbent assay (ELISA). Plates were coated with recombinant protein OspC 1µg/ml and incubated with specific mouse sera collected at timepoint 42 days pre-challenge. Sera were diluted to 218700 times.

Supplementary Figure 2 Specific total IgG titers were measured in an Enzyme-linked immunosorbent assay (ELISA). Plates were coated with recombinant protein (TSLPI, Salp15, tHRF, Tix-5 1µg/ml) and incubated with mouse sera collected at timepoint 42 days pre-challenge. Sera were diluted to 218700 times.
