# Supplementary figures and images for: Tick-Tattoo: DNA Vaccination Against B. burgdorferi or Ixodes scapularis Tick Proteins
Source: Front Immunol. 2021 Feb 25;12:615011. doi: 10.3389/fimmu.2021.615011 (PMC7946838; doi:10.3389/fimmu.2021.615011)

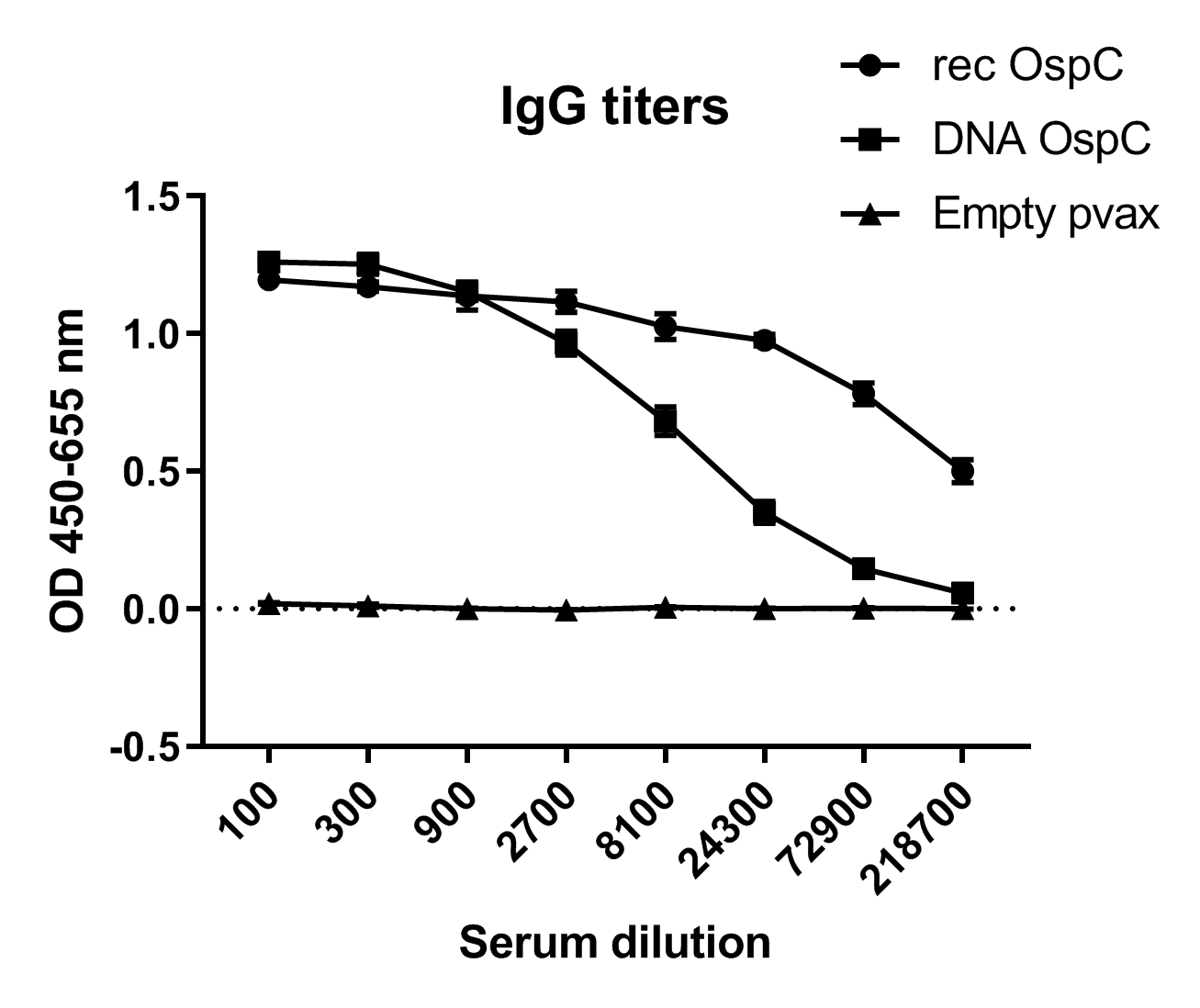

Supplement: Supplementary Figure 1 — Specific total IgG titers were measured in an Enzyme-linked immunosorbent assay (ELISA). Plates were coated with recombinant protein OspC 1µg/ml and incubated with specific mouse sera collected at timepoint 42 days pre-challenge. Sera were diluted to 218700 times. [file Image_1.tif]

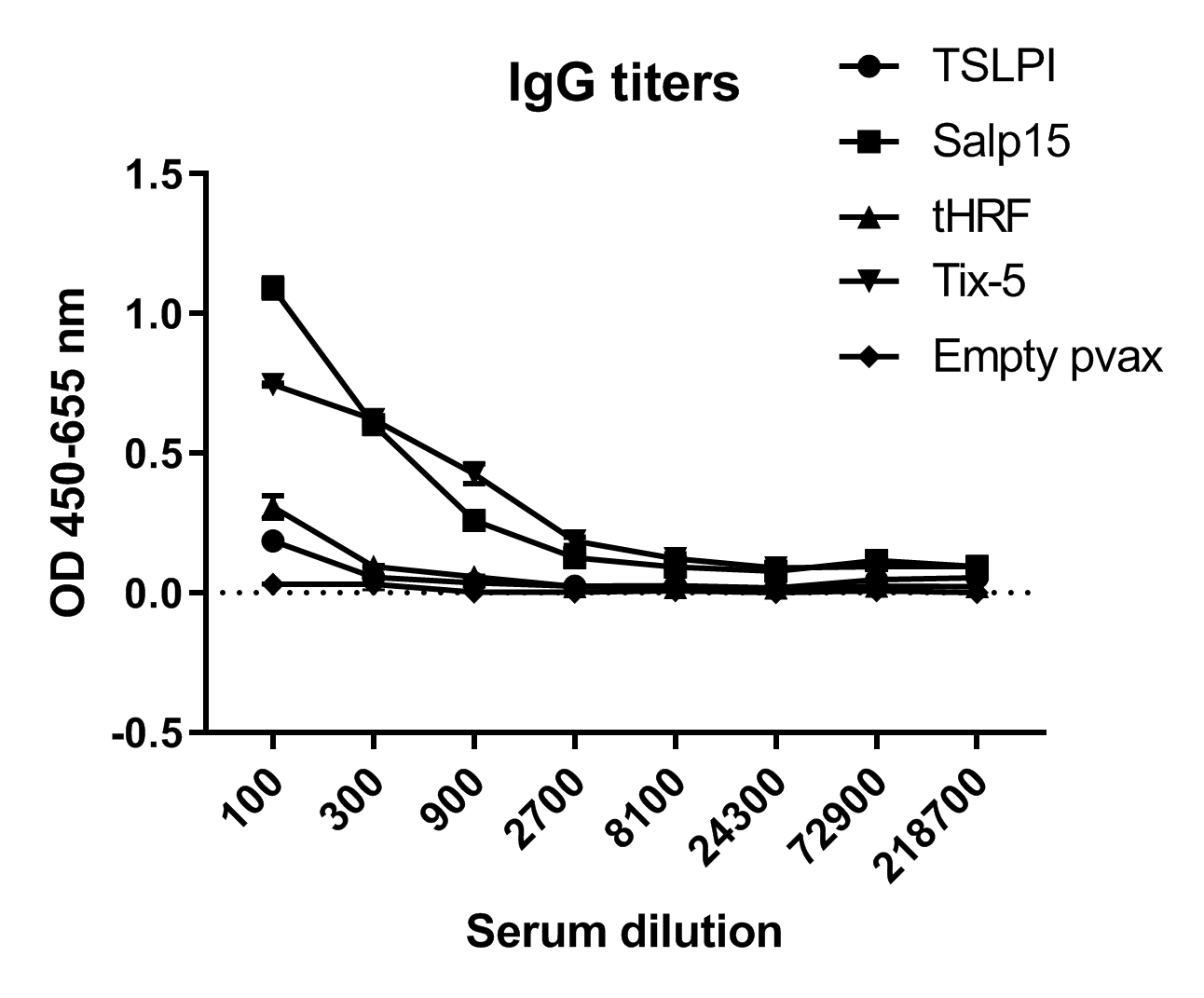

Supplement: Supplementary Figure 2 — Specific total IgG titers were measured in an Enzyme-linked immunosorbent assay (ELISA). Plates were coated with recombinant protein (TSLPI, Salp15, tHRF, Tix-5 1µg/ml) and incubated with mouse sera collected at timepoint 42 days pre-challenge. Sera were diluted to 218700 times. [file Image_2.tif]
